# Computational Effective Fault Detection by Means of Signature Functions

**DOI:** 10.1371/journal.pone.0150787

**Published:** 2016-03-07

**Authors:** Przemyslaw Baranski, Piotr Pietrzak

**Affiliations:** 1 Institute of Electronics, Lodz University of Technology, Wolczanska 211/215, Lodz, Poland; 2 Department of Microelectronics and Computer Science, Lodz University of Technology, Wolczanska 221/223, Lodz, Poland; Chongqing University, CHINA

## Abstract

The paper presents a computationally effective method for fault detection. A system’s responses are measured under healthy and ill conditions. These signals are used to calculate so-called signature functions that create a signal space. The current system’s response is projected into this space. The signal location in this space easily allows to determine the fault. No classifier such as a neural network, hidden Markov models, etc. is required. The advantage of this proposed method is its efficiency, as computing projections amount to calculating dot products. Therefore, this method is suitable for real-time embedded systems due to its simplicity and undemanding processing capabilities which permit the use of low-cost hardware and allow rapid implementation. The approach performs well for systems that can be considered linear and stationary. The communication presents an application, whereby an industrial process of moulding is supervised. The machine is composed of forms (dies) whose alignment must be precisely set and maintained during the work. Typically, the process is stopped periodically to manually control the alignment. The applied algorithm allows on-line monitoring of the device by analysing the acceleration signal from a sensor mounted on a die. This enables to detect failures at an early stage thus prolonging the machine’s life.

## Nomenclature

As scientific texts use different notations for vectors, matrices, random variables, derivatives with respect to vectors, Table 1 provides the convention applied in this paper.

**Table 1:** The list of symbols and notations used in this paper.

Symbol	Description
*σ*	standard deviation
*σ*^2^	variance, second central moment
*E*{ ⋅ }	expectation; average value; expected value; first moment
*x*, *X*	scalar values
*f*(*t*)	one dimensional signal
〈*f*(*t*), *g*(*t*)〉	dot (inner or scalar) product of two signals; $\langle f(t),g(t)\rangle =\int_{T_{0}}^{T_{1}}f\left(t\right)\;g\left(t\right)\;dt$
*r* × *c*	size of a matrix: *r* rows and *c* columns
**A**	matrix—bold, capital letter
**x**	column vector—bold, small letter;**x** = [*x*_1_, *x*_2_, …, *x*_*m*_]^*T*^
**f**(*t*)	column vector of one dimensional signals—bold, small letter;**f**(*t*) = [*f*_1_(*t*), *f*_2_(*t*), …, *f*_*m*_(*t*)]^*T*^
$\frac{\partial f(x)}{\partial x}$	derivative of function *f*(**x**) with respect to column vector **x**; denominator layout; $\frac{\partial f\left(x\right)}{\partial x}={\left[\frac{\partial f\left(x\right)}{\partial x_{1}},\frac{\partial f\left(x\right)}{\partial x_{2}},\ldots ,\frac{\partial f\left(x\right)}{\partial x_{m}}\right]}^{T}$

## Introduction

There are many applications that require on-line fault diagnosis, e.g. to monitor the quality of manufactured products or to improve the reliability and safety of a system.

The literature provides many sophisticated solutions that are, in most cases, computationally demanding, hence problematic to implement in embedded devices where processing power is restricted. Moreover, many scenarios require a strictly limited processing time. Computationally effective solutions can be implemented using simple and low-cost hardware which also cuts development time and overall cost.

The article presents a method for detecting faults by analysing the time responses. The solution was applied in monitoring a moulding process which is commonly used in industry to manufacture objects from pliable material. A moulding device is typically composed of two forms whose inner shape determine the outline of a manufactured object. At the beginning of the process, the dies are shifted towards and pressed against each other with huge force to assure no inbetween gaps. The raw material is injected into the forms which are then detached to eject the solidified object. The proper alignment of the dies is crucial to the process. When the contact planes of the dies are skewed, the impact causes uneven wear-off of the forms and their untimely deterioration. Therefore, the production process must be stopped every now and again to measure the forms’ alignment and recalibrate if need be. A reliable and computationally inexpensive method for automatic detection of the dies’ adjustment is desirable in industry.

The suggested approach makes use of an acceleration signal from the sensor to detect a faulty moulding device. In the application, the response signal can be considered as stationary and can also be analysed solely in the time domain by using the dot (inner, scalar) product whose computational cost is proportional to the number of samples. There is no need to employ transforms such as wavelet or Fourier, as in other approaches. This greatly reduces computation complexity and allows to use simpler and lower-priced hardware. This method does not require classifiers such as a neural network, support vector machine (SVM), etc.

The subject of controlling the dies of a moulding device is not widely described in the literature. However, similar challenges arise in different applications. The excellent paper [1] shows an interesting application whereby a stamping process is monitored for a missing part in the production line. The tonnage signal is analysed with help of the recurring plot (RP) technique [2] which is used to capture a fine deviation of a non-stationary signal. The computation of the presented two dimensional RP matrix is fairly burdensome. The classification is determined on the base of the difference between the calculated RP matrix and a reference one. The overall computation cost is proportional to the squared number of analysed samples (being around several hundreds in the considered application).

The comprehensive work [3] describes an application of detecting several faults in a stamping process. The sampled strain signal is transformed using wavelets to acquire a vector of coefficients. The approach necessitates a classifier to make a decision about the fault. The authors compare the effectiveness of hidden Markov models (HMM), artificial neural network (ANN), support vector machine (SVM), support vector regression (SVR) and a proprietary classifier. The optimal training of a classifier is a problem of its own. The approach is suitable to analyse non-stationary signals with a poor signal-to-noise ratio (SNR), however it is too computationally demanding to be implemented in a microcontroller.

A similar work is also reported in [4] where a number *p* of autoregressive coefficients need to be computed. Thus the computation cost is *p* (being at least 4) times the cost of calculating the dot product. Moreover, the classifier based on HMM needs to be trained.

The approach presented in [5] analyses the tonnage signal from a stamping process with the help of wavelet transform and a fairly complex technique called statistical process control (SPC).

Similar solutions are employed to prevent the damage of mechanical systems or warn of a fault, e.g. in engines [6], pumps [7], gear-boxes [8], wind turbines [9], etc.

The typical approach to fault diagnosis is composed of the following three stages:

1. signal transformation—the output response (or responses) of a device or system is often decomposed by Fourier, wavelet [10–12], (short time Fourier transform) STFT, Wiegner-Ville [13], EEMD (empirical mode decomposition) [7] transforms to acquire a vector of parameters;
2. reduction of the vector size—the vector contains many irrelevant features that can be neglected to simplify the classification problem. The following methods are commonly used in fault diagnosis: principal component analysis (PCA) [14], kernel entropy analysis (KECA) [11], kernel principal component analysis [11, 15], uncorrelated multilinear PCA, uncorrelated multilinear PCA [16] and others;
3. classification—the reduced vector is an input to a neural network to perform fault classification. As a neural network can produce not deterministic results [17, 18] due to overlearning and local extrema, often the support vector machine (SVM) is used, as in [10, 19]. The work [7] uses Bayesian network and shows its superiority over neural network or SVM in an application of monitoring a gear pump. Some works also use hidden Markov models, as in [3, 4].

The above-presented methodology is general, flexible and allows to analyse signals produced by non-stationary and non-linear systems. This comprehensiveness, however, incurs computational complexity that in most cases precludes implementation in embedded devices. At the same time, there are systems that can be considered stationary and linear and whose output responses for different faults are distinguishable in the time domain. Therefore, there is no need to apply signal transform which imposes a considerable computational burden. The approach suggested in this paper does not require a complex classifier, such as a neural network, HMM, SVM, etc. The system’s response is projected into the space created by the so-called signature functions, which are computed from the training responses. The location of the signal in that space determines the fault type. The properties of the presented method make the approach suitable for embedded industrial solutions where hardware cost, reliability and simplicity are important factors.

## Materials and Methods

### Derivation

In this section, we will develop a method for determining the system’s state basing on its time response. Thus, the responses pertaining to different conditions should be distinguishable in the time domain. This implies that the analysed system should behave as stationary and linear.

First, the responses corresponding to different conditions of the system need to be acquired. This constitutes training data for the method. Let *a*_*i*_(*t*), where *i* ranges from 1 to *A*, denote the system’s responses measured in its in healthy condition. Let *b*_*i*_(*t*), where *i* ranges from 1 to *B*, represent the system’s responses under the first type of malfunctioning. Let *c*_*i*_(*t*), where *i* ranges from 1 to *C*, represent the system’s response under the second type of malfunctioning. By analogy, we can define other functions representing the system’s responses under other types of malfunctioning. The total number of states (healthy and malfunctioning) is denoted by *ξ*. The total number of time responses is

$$\begin{array}{c} \Theta =A+B+C+\ldots \end{array}$$ (1)

For the sake of compactness, we arrange the training signals in the following vector of functions

$$\begin{array}{c} f\left(t\right)={\left[a_{1}\left(t\right),\ldots ,a_{A}\left(t\right),b_{1}\left(t\right),\ldots ,b_{B}\left(t\right),c_{1}\left(t\right),\ldots \right]}^{T} \end{array}$$ (2)

whose dimension is Θ × 1.

On the base of the training signals, we want to calculate so-called signature functions. Let $\hat{a}\left(t\right)$ denote the signature of the healthy state. We demand, that $\hat{a}\left(t\right)$ be similar to signals *a*_*i*_(*t*) (*i* = 1…*A*) and at the same time unrelated (orthogonal) to signals *b*_*i*_(*t*), *c*_*i*_(*t*), …. Consequently, signature function $\hat{b}\left(t\right)$ should be similar to signals *b*_*i*_(*t*) and orthogonal to signals *a*_*i*_(*t*), *c*_*i*_(*t*), …This simplifies greatly the classification problem. The current response *h*(*t*) of the system is registered and compared to signature functions $\hat{a}\left(t\right)$, $\hat{b}\left(t\right)$, …Then, for instance, in the healthy state, *h*(*t*) bears a resemblance to $\hat{a}\left(t\right)$ and is orthogonal to other signature functions.

We assume that the signatures can be expressed as linear combinations of the training signals, hence can be written as

$$\begin{array}{c} \hat{a}\left(t\right)=f{\left(t\right)}^{T}x_{a} \end{array}$$ (3)

$$\begin{array}{c} \hat{b}\left(t\right)=f{\left(t\right)}^{T}x_{b} \end{array}$$ (4)

$$\begin{array}{rr} \hat{c}\left(t\right) & =f{\left(t\right)}^{T}x_{c}\\ \vdots & \end{array}$$ (5)

where unknown vectors **x**_*a*_, **x**_*b*_, **x**_*c*_, …are of dimension Θ × 1.

The challenge is to compute signature functions, which is equivalent to calculating vectors **x**_*a*_, **x**_*b*_, …so that the signatures are robust against noise and provide minimum classification error for unseen data. We will come back to this problem later.

Another issue is to measure the resemblance between two signals. For that purpose, various techniques are employed, e.g. the sum of squared differences, the sum of absolute differences, dynamic time warping.

For our purpose, we employed the dot (inner, scalar) product, which is a linear operation. Let 〈*g*(*t*), *h*(*t*)〉 denote the dot product of two functions, defined here by

$$\langle g\left(t\right),h\left(t\right)\rangle =\int_{T_{0}}^{T_{1}}g\left(t\right)\;h\left(t\right)\text{ d}t$$ (6)

where (*T*_0_, *T*_1_) denotes the time range of the relevant system’s response.

Let *h*(*t*) denote the system’s response. If the system is healthy then

$$\begin{array}{c} c_{a}=\langle \hat{a}\left(t\right),h\left(t\right)\rangle \approx 1, \end{array}$$ (7)

$$\begin{array}{c} c_{b}=\langle \hat{b}\left(t\right),h\left(t\right)\rangle \approx 0, \end{array}$$ (8)

$$\begin{array}{c} c_{c}=\langle \hat{c}\left(t\right),h\left(t\right)\rangle \approx 0,\;\text{etc}. \end{array}$$ (9)

When the system exhibits a fault of the first type, then

$$\begin{array}{c} c_{a}=\langle \hat{a}\left(t\right),h\left(t\right)\rangle \approx 0, \end{array}$$ (10)

$$\begin{array}{c} c_{b}=\langle \hat{b}\left(t\right),h\left(t\right)\rangle \approx 1, \end{array}$$ (11)

$$\begin{array}{c} c_{a}=\langle \hat{c}\left(t\right),h\left(t\right)\rangle \approx 0,\;\text{etc}. \end{array}$$ (12)

By analogy, in case of the second type of failure

$$\begin{array}{c} c_{a}=\langle \hat{a}\left(t\right),h\left(t\right)\rangle \approx 0, \end{array}$$ (13)

$$\begin{array}{c} c_{b}=\langle \hat{b}\left(t\right),h\left(t\right)\rangle \approx 0, \end{array}$$ (14)

$$\begin{array}{c} c_{a}=\langle \hat{c}\left(t\right),h\left(t\right)\rangle \approx 1,\;\text{etc}. \end{array}$$ (15)

**Fig 1:**
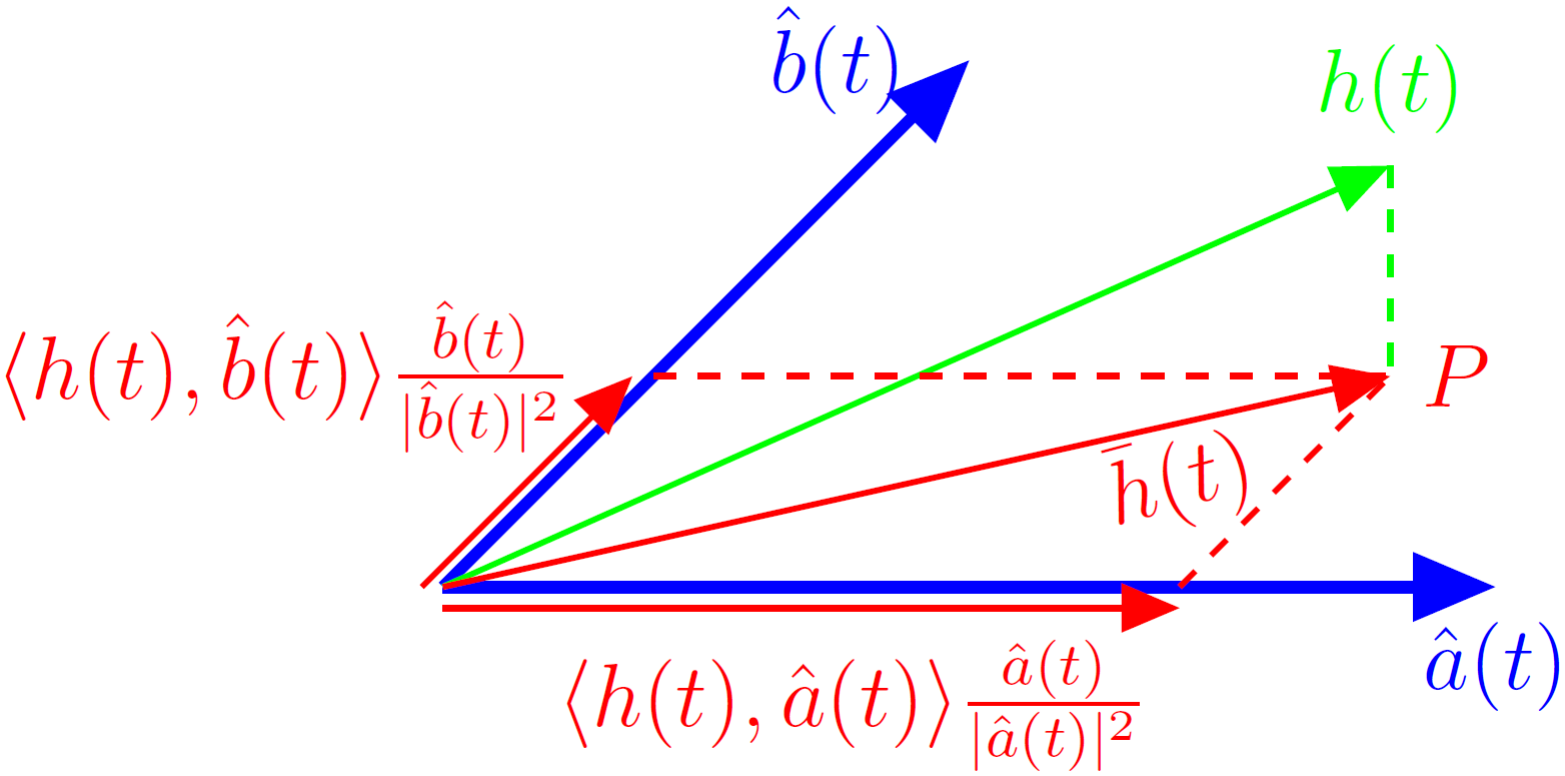
An example of projecting a signal *h*(*t*) on the two-dimensional space spanned by two signals $\hat{a}\left(t\right)$ and $\hat{b}\left(t\right)$. The projection of *h*(*t*) reads $\bar{h}\left(t\right)$. The coordinates of the head of this vector are denoted by *P*.

In other words, a signal space is spanned on the signature functions. The location of the system’s response *h*(*t*) determines its state. Fig 1 shows an example of projecting an analysed signal *h*(*t*) on the plane spanned by two signature functions $\hat{a}\left(t\right)$ and $\hat{b}\left(t\right)$. The projection of *h*(*t*) reads $\bar{h}\left(t\right)$. This vector has the following coordinates in the considered signal space

$$\begin{array}{c} \left[\begin{array}{c} c_{a}\\ c_{b} \end{array}\right]=\left[\begin{array}{c} \langle \hat{a}\left(t\right),h\left(t\right)\rangle \\ \langle \hat{b}\left(t\right),h\left(t\right)\rangle \end{array}\right]\;. \end{array}$$ (16)

Signal *h*(*t*) can also be considered as a point whose coordinates in the signal plane read

$$\begin{array}{c} P\left(h(t)\right)=\left(c_{a},c_{b}\right)=\left(\langle \hat{a}\left(t\right),h\left(t\right)\rangle ,\langle \hat{b}\left(t\right),h\left(t\right)\rangle \right)\;. \end{array}$$ (17)

Given the system’s response *h*(*t*) and calculated coordinates *P*(*h*(*t*)) in the signal space, the problem of state classification is then easy. Checking the location of *P*(*h*(*t*)) with reference to the decision boundaries is straightforward. An example criterion can be based on the maximum value of *c*_*a*_, *c*_*b*_, *c*_*c*_, ….

As indicated earlier, the challenge is to determine the signature functions $\hat{a}\left(t\right)$, $\hat{b}\left(t\right)$, …that is to calculate vectors **x**_*a*_, **x**_*b*_, …of unknown coefficients—see Eqs (3–5). The resultant signatures should be resilient against noise and provide a minimum misclassification rate for unseen signals.

**Fig 2:**
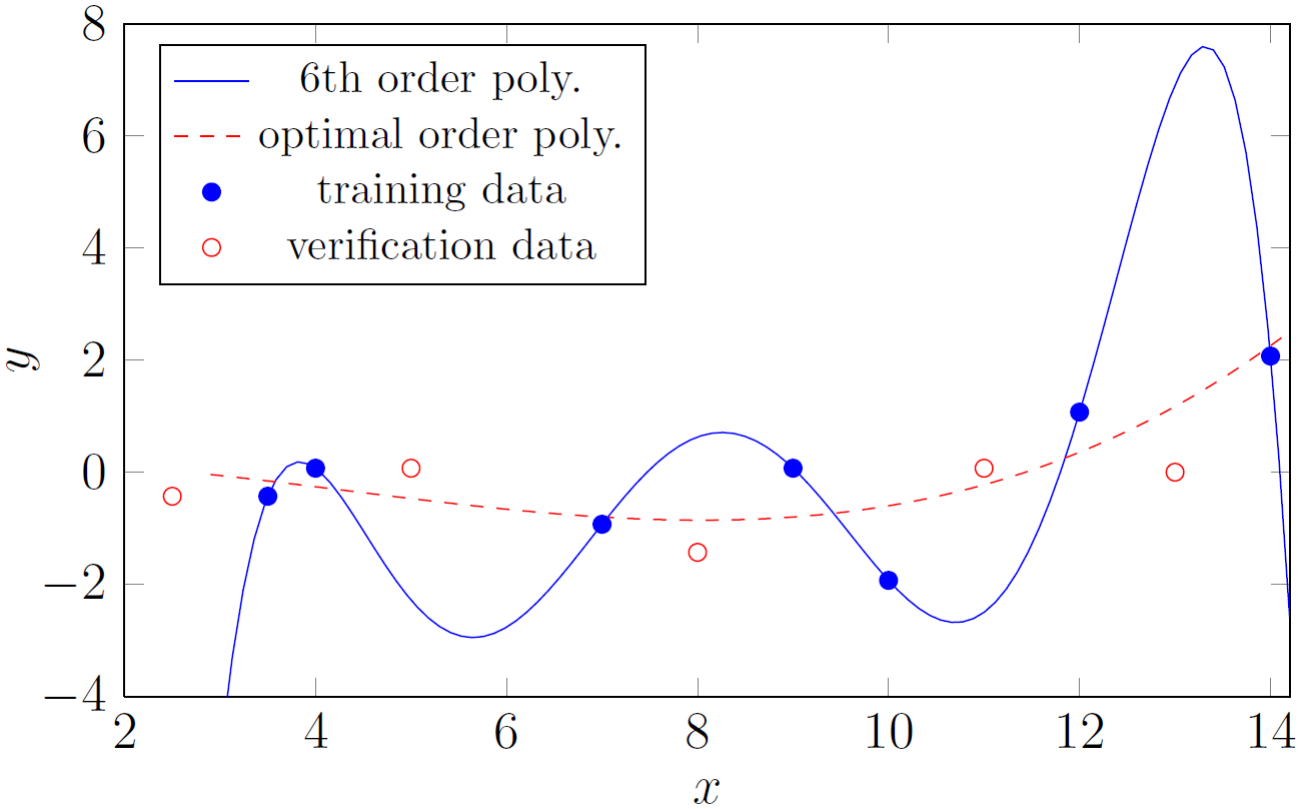
Illustration of overfitting. Blue bullets—training data; red circles “unseen” (verification) data; continuous blue line—polynomial fitted accurately to 7 training points; red dashed lined—polynomial of optimal order fitted to the training data.

The task is similar to regression analysis, whereby a curve equation is calculated on the base of the training data. When the underlying physical model is unknown, the problem is difficult. The curve should provide a minimum error for unseen data. Overfitting the curve to the training data results in a poor fit to the unseen data—this is illustrated in Fig 2. This problem is discussed in detail in [20] (especially Examples 4.3 and 4.4) and in [21]. In the case of neural networks, this phenomenon is described as overlearning.

Calculating the signature functions in the way presented below leads to overfitting to the training data. However, this is an instructive step, as it suggests a solution. Demanding for $\hat{a}\left(t\right)$

$$\begin{array}{c} \begin{array}{cccc} \langle a_{1}\left(t\right),\hat{a}\left(t\right)\rangle & = & \langle a_{1}\left(t\right),f{\left(t\right)}^{T}x_{a}\rangle & =1\\ \vdots & & \vdots & \vdots \\ \langle a_{A}\left(t\right),\hat{a}\left(t\right)\rangle & = & \langle a_{A}\left(t\right),f{\left(t\right)}^{T}x_{a}\rangle & =1\\ \langle b_{1}\left(t\right),\hat{a}\left(t\right)\rangle & = & \langle b_{1}\left(t\right),f{\left(t\right)}^{T}x_{a}\rangle & =0\\ \vdots & & \vdots & \vdots \\ \langle c_{1}\left(t\right),\hat{a}\left(t\right)\rangle & = & \langle b_{B}\left(t\right),f{\left(t\right)}^{T}x_{a}\rangle & =0\\ \vdots & & \vdots & \vdots \end{array} \end{array}$$ (18)

and using the linearity of the 〈·, ·〉 operator, leads to the following matrix equation

$$\begin{array}{c} D\;x_{a}=y_{a} \end{array}$$ (19)

where $y_{a}={\left[\overset{\Theta }{\overset{︷}{\underset{A}{\underset{︸}{1,\ldots ,1}},0,\ldots ,0}}\right]}^{T}$ is a Θ × 1 vector and **D** is the following symmetric, Θ × Θ matrix

$$\begin{array}{c} D=\langle f\left(t\right),f{\left(t\right)}^{T}\rangle =\left[\begin{array}{ccccc} \langle a_{1}\left(t\right),a_{1}\left(t\right)\rangle & \ldots & \langle a_{1}\left(t\right),a_{A}\left(t\right)\rangle & \langle a_{1}\left(t\right),b_{1}\left(t\right)\rangle & \ldots \\ \langle a_{2}\left(t\right),a_{1}\left(t\right)\rangle & \ldots & \langle a_{2}\left(t\right),a_{A}\left(t\right)\rangle & \langle a_{2}\left(t\right),b_{1}\left(t\right)\rangle & \ldots \\ \vdots & & \vdots & \vdots & \\ \langle b_{1}\left(t\right),a_{1}\left(t\right)\rangle & \ldots & \langle b_{1}\left(t\right),a_{A}\left(t\right)\rangle & \langle b_{1}\left(t\right),b_{1}\left(t\right)\rangle & \ldots \\ \vdots & & \vdots & \vdots & \end{array}\right]\;. \end{array}$$ (20)

Then

$$\begin{array}{c} x_{a}=D^{-1}y_{a} \end{array}$$ (21)

and

$$\begin{array}{c} \hat{a}\left(t\right)=f{\left(t\right)}^{T}x_{a}\;. \end{array}$$ (22)

**Fig 3:**
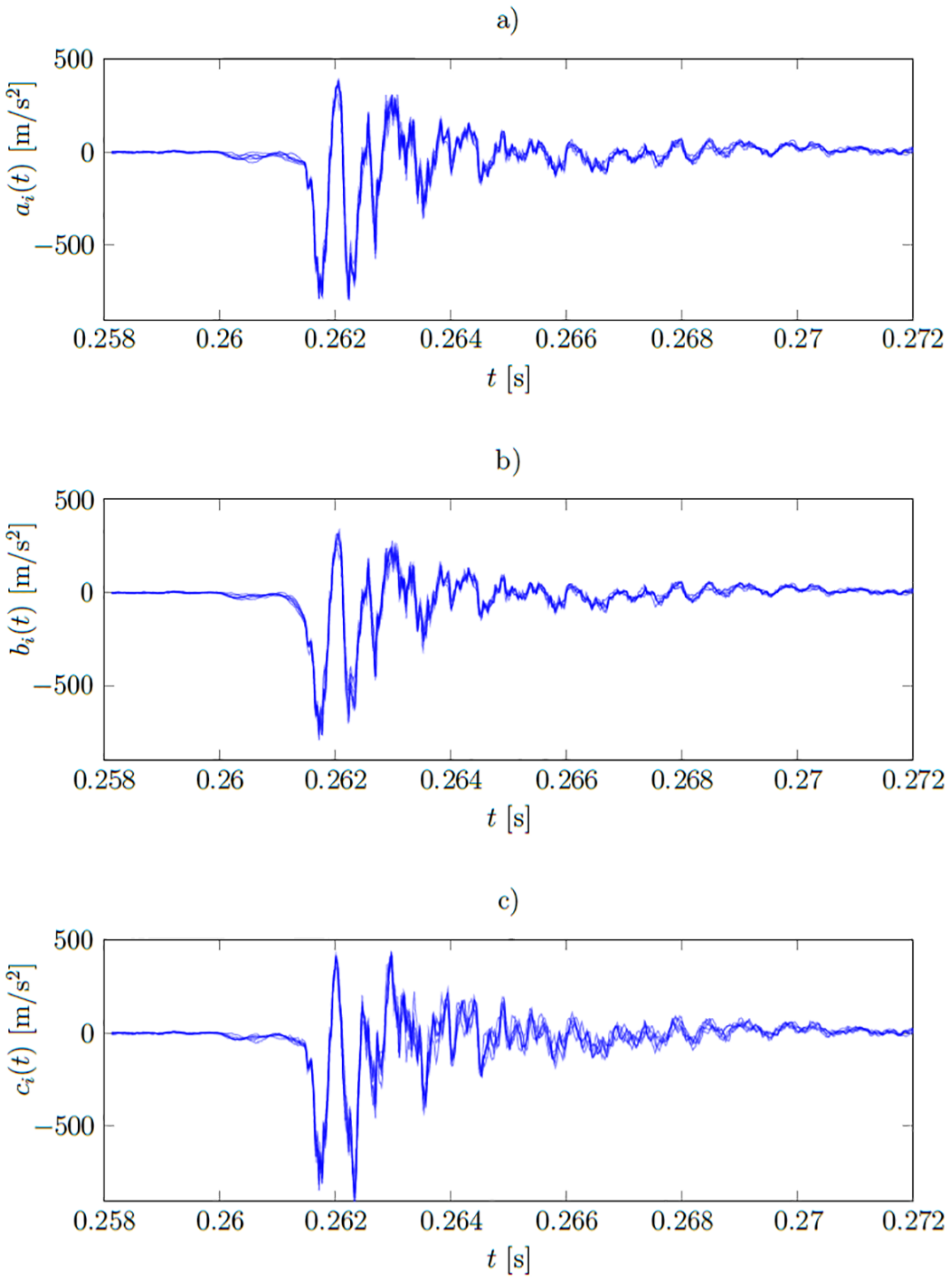
Plots of functions from the training set. a) *a*_*i*_(*t*)—signals measured in healthy state (*i* = 1…6); b) *b*_*i*_(*t*)—first type fault (*i* = 1…6); c) *c*_*i*_(*t*)—second type fault (*i* = 1…6).

**Fig 4:**
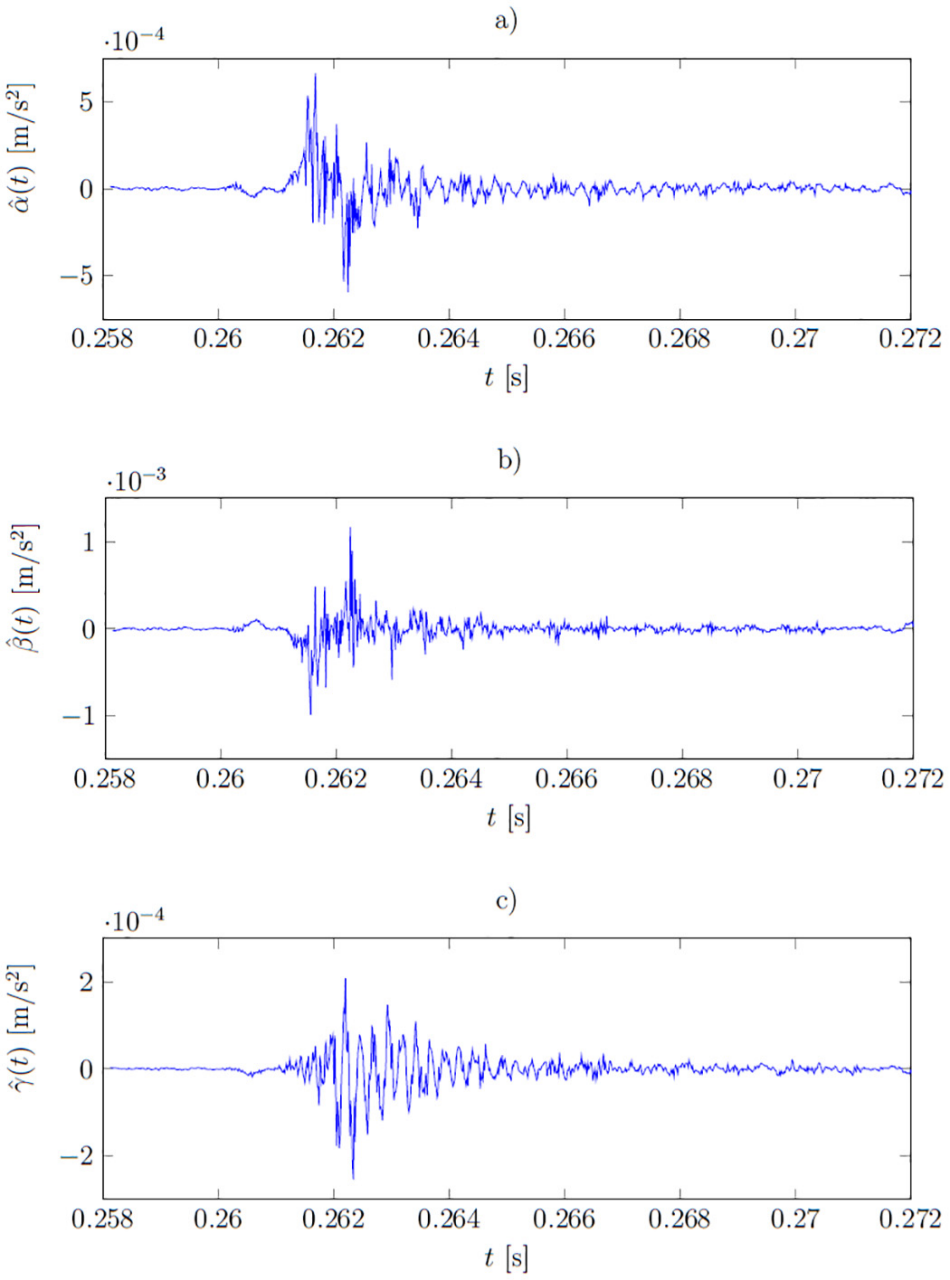
Plots of the overfitted signature functions (calculated on the base of the training set **f**(*t*)). a) under healthy conditions $\hat{\alpha }\left(t\right)$, b) under the first type fault $\hat{\beta }\left(t\right)$ and c) under the second type fault $\hat{\gamma }\left(t\right)$.

The signature function $\hat{a}\left(t\right)$ calculated in this way is perfectly fitted to the training signals. Fig 3a, 3b and 3c present example training signals *a*_*i*_(*t*), *b*_*i*_(*t*), *c*_*i*_(*t*) and Fig 4a, 4b and 4c corresponding signatures which are noisy. The interpretation of the shape of these signals is difficult. Moreover, the signatures should assume non-zero values only where the training signals representing different states differ. For example, the training data representing healthy and ill states in Fig 3 start to differ only at around *t* ≈ 0.2612 *s*, however the overfitted signatures are of significant values already at *t* ≈ 0.2605 *s*. This causes a poor fit to the unseen data and is illustrated later in the Result section.

To mitigate this problem, we need to constrain the signatures to take non-zero values, solely where the signals representing different states are different.

We demand the subsequent orthogonality conditions

$$\begin{array}{c} E\left\{\langle \hat{a}\left(t\right),a_{i}\left(t\right)\rangle \right\}=1\;\text{for}\;i=1\ldots A \end{array}$$ (23)

$$\begin{array}{c} E\left\{\langle \hat{a}\left(t\right),b_{i}\left(t\right)\rangle \right\}=0\;\text{for}\;i=1\ldots B \end{array}$$ (24)

$$\begin{array}{c} E\left\{\langle \hat{a}\left(t\right),c_{i}\left(t\right)\rangle \right\}=0\;\text{for}\;i=1\ldots C\\ \vdots \end{array}$$ (25)

$$\begin{array}{c} E\left\{\langle \hat{b}\left(t\right),a_{i}\left(t\right)\rangle \right\}=0\;\text{for}\;i=1\ldots A \end{array}$$ (26)

$$\begin{array}{c} E\left\{\langle \hat{b}\left(t\right),b_{i}\left(t\right)\rangle \right\}=1\;\text{for}\;i=1\ldots B \end{array}$$ (27)

$$\begin{array}{c} E\left\{\langle \hat{b}\left(t\right),c_{i}\left(t\right)\rangle \right\}=0\;\text{for}\;i=1\ldots C\\ \vdots \end{array}$$ (28)

where E{ ⋅ } denotes expectation (average value). This constraint prevents the signature functions fit the training data perfectly. Instead, the average residual error equals 0. This mitigates the influence of noise on fitting. Defining the vectors

$$\begin{array}{c} e_{a}=\frac{1}{A}\left[\begin{array}{c} \langle a_{1}\left(t\right),a_{1}\left(t\right)\rangle +\ldots +\langle a_{1}\left(t\right),a_{A}\left(t\right)\rangle \\ \vdots \\ \langle a_{A}\left(t\right),a_{1}\left(t\right)\rangle +\ldots +\langle a_{A}\left(t\right),a_{A}\left(t\right)\rangle \\ \langle b_{1}\left(t\right),a_{1}\left(t\right)\rangle +\ldots +\langle b_{1}\left(t\right),a_{A}\left(t\right)\rangle \\ \vdots \\ \langle b_{B}\left(t\right),a_{1}\left(t\right)\rangle +\ldots +\langle b_{B}\left(t\right),a_{A}\left(t\right)\rangle \\ \langle c_{1}\left(t\right),a_{1}\left(t\right)\rangle +\ldots +\langle c_{1}\left(t\right),a_{A}\left(t\right)\rangle \\ \vdots \end{array}\right] \end{array}$$ (29)

$$\begin{array}{c} e_{b}=\frac{1}{B}\left[\begin{array}{c} \langle a_{1}\left(t\right),b_{1}\left(t\right)\rangle +\ldots +\langle a_{1}\left(t\right),b_{B}\left(t\right)\rangle \\ \vdots \\ \langle a_{A}\left(t\right),b_{1}\left(t\right)\rangle +\ldots +\langle a_{A}\left(t\right),b_{B}\left(t\right)\rangle \\ \langle b_{1}\left(t\right),b_{1}\left(t\right)\rangle +\ldots +\langle b_{1}\left(t\right),b_{M}\left(t\right)\rangle \\ \vdots \\ \langle b_{M}\left(t\right),b_{1}\left(t\right)\rangle +\ldots +\langle b_{M}\left(t\right),b_{M}\left(t\right)\rangle \end{array}\right] \end{array}$$ (30)

$$\begin{array}{c} e_{c}=\frac{1}{C}\left[\begin{array}{c} \langle a_{1}\left(t\right),c_{1}\left(t\right)\rangle +\ldots +\langle a_{1}\left(t\right),c_{C}\left(t\right)\rangle \\ \vdots \\ \langle a_{A}\left(t\right),c_{1}\left(t\right)\rangle +\ldots +\langle a_{A}\left(t\right),c_{C}\left(t\right)\rangle \\ \vdots \end{array}\right] \end{array}$$ (31)

whose dimensions are Θ × 1, the conditions (23–25) can be expressed by

$$\begin{array}{c} \left[\begin{array}{c} e_{a}^{T}\\ e_{b}^{T}\\ e_{c}^{T}\\ \vdots \end{array}\right]x_{a}=\left[\begin{array}{c} 1\\ 0\\ 0\\ \vdots \end{array}\right] \end{array}$$ (32)

or

$$\begin{array}{c} E^{T}x_{a}=v_{a} \end{array}$$ (33)

where **E** = [**e**_*a*_, **e**_*b*_, **e**_*c*_, …] is of size Θ × *ξ* and **v**_*a*_ = [1, 0, 0, …]^*T*^ is of size *ξ* × 1. By the same vein, conditions (26–28) can be written as

$$\begin{array}{c} E^{T}x_{b}=v_{b} \end{array}$$ (34)

where **v**_*b*_ = [0, 1, 0, …]^*T*^. Vectors **x**_*a*_, **x**_*b*_, etc. cannot simply be calculated from Eqs (33 and 34) which pose an under-determined set of equations (**E** is a non-square matrix). We also impose the following minimum energy constraints

$$\begin{array}{c} \langle \hat{a}\left(t\right),\hat{a}\left(t\right)\rangle =\text{min} \end{array}$$ (35)

$$\begin{array}{c} \langle \hat{b}\left(t\right),\hat{b}\left(t\right)\rangle =\text{min}\\ \vdots \end{array}$$ (36)

which have the beneficial effect of smoothing the signature functions. This phenomenon is described as “the blessing of smoothness” and improves the prediction capabilities for unseen data, as explained in [21]. This constraint plays an important role—the signatures will assume non-zero values, only where the training data corresponding to different states bear differences.

Hence, the problem is to minimize $\langle \hat{a}\left(t\right),\hat{a}\left(t\right)\rangle$ subject to Eqs (23–25). Minimizing $\langle \hat{b}\left(t\right),\hat{b}\left(t\right)\rangle$ subject to Eqs (26–28) is separable from the previous problem. We will use the method of Lagrange multipliers [22] and form the following Lagrangian

$$\begin{array}{cc} L_{a}\left(x_{a},\lambda_{a}\right) & =\langle \hat{a}\left(t\right),\hat{a}\left(t\right)\rangle +2\cdot \lambda_{a1}\left(E\left\{\langle \hat{a}\left(t\right),a_{i}\left(t\right)\rangle \right\}-1\right)+\\ & \;+2\cdot \lambda_{a2}\left(E\left\{\langle \hat{a}\left(t\right),b_{i}\left(t\right)\rangle \right\}\right)+\\ & \;+2\cdot \lambda_{a3}\left(E\left\{\langle \hat{a}\left(t\right),c_{i}\left(t\right)\rangle \right\}\right)+\ldots \end{array}$$ (37)

where *λ*_*a*_ = [*λ*_*a*1_, *λ*_*a*2_, …]^*T*^ is the vector of Lagrange multipliers. The derivative

$$\begin{array}{c} \frac{\partial }{\partial x_{a}}L\left(x_{a},\lambda_{a}\right)=\left[\begin{array}{c} \frac{\partial }{\partial x_{a1}}L\left(x_{a},\lambda_{a}\right)\\ \frac{\partial }{\partial x_{a2}}L\left(x_{a},\lambda_{a}\right)\\ \vdots \end{array}\right] \end{array}$$ (38)

should vanish implying that the minimum of $\langle \hat{a}\left(t\right),\hat{a}\left(t\right)\rangle$ was met and the conditions (23–25) satisfied.

The derivatives of conditions (35 and 36), as can be shown by direct computation, read

$$\begin{array}{c} \frac{\partial }{\partial x_{a}}\langle \hat{a}\left(t\right),\hat{a}\left(t\right)\rangle =2D\;x_{a} \end{array}$$ (39)

$$\begin{array}{c} \frac{\partial }{\partial x_{b}}\langle \hat{b}\left(t\right),\hat{b}\left(t\right)\rangle =2D\;x_{b}\\ \vdots \end{array}$$ (40)

Differentiating conditions (23–25) yields

$$\begin{array}{c} \frac{\partial }{\partial x_{a}}E\left\{\langle \hat{a}\left(t\right),a_{i}\left(t\right)\rangle \right\}=e_{a} \end{array}$$ (41)

$$\begin{array}{c} \frac{\partial }{\partial x_{a}}E\left\{\langle \hat{a}\left(t\right),b_{i}\left(t\right)\rangle \right\}=e_{b} \end{array}$$ (42)

$$\begin{array}{c} \frac{\partial }{\partial x_{a}}E\left\{\langle \hat{a}\left(t\right),c_{i}\left(t\right)\rangle \right\}=e_{c}\\ \vdots \end{array}$$ (43)

Hence

$$\begin{array}{c} \frac{\partial }{\partial x_{a}}L\left(x_{a},\lambda_{a}\right)=2Dx_{a}+2E\lambda_{a}=0 \end{array}$$ (44)

$$\begin{array}{rr} \frac{\partial }{\partial x_{b}}L\left(x_{b},\lambda_{b}\right)=2Dx_{b}+2E\lambda_{b} & =0\\ \vdots & \end{array}$$ (45)

Thus, to calculate **x**_*a*_, the set of two matrix Eqs (33 and 44) needs to be solved. For the sake of readability, we rewrite these equations

$$\begin{array}{c} E^{T}x_{a}=v_{a} \end{array}$$ (46)

$$\begin{array}{c} 2Dx_{a}+2E\lambda_{a}=0 \end{array}$$ (47)

where **E**^*T*^ is a non-square matrix of size Θ × *ξ*; **D** is a square matrix of size Θ × Θ. **x**_*a*_ cannot be determined directly from Eq (46) by inverting the non-square matrix **E**^*T*^. Instead, solving for **x**_*a*_ in Eq (47) yields

$$\begin{array}{c} x_{a}=-D^{-1}E\lambda_{a}\;. \end{array}$$ (48)

Substituting this to Eq (46) and calculating *λ*_*a*_ leads to

$$\begin{array}{c} \lambda_{a}=-{\left(E^{T}D^{-1}E\right)}^{-1}v_{a}\;. \end{array}$$ (49)

Plugging *λ*_*a*_ back to Eq (47) finally results in

$$\begin{array}{c} x_{a}=D^{-1}E{\left(E^{T}D^{-1}E\right)}^{-1}v_{a}\;. \end{array}$$ (50)

The function $\hat{a}\left(t\right)$ can now be calculated from Eq (3) or from

$$\begin{array}{c} \hat{a}\left(t\right)=f{\left(t\right)}^{T}D^{-1}E{\left(E^{T}D^{-1}E\right)}^{-1}v_{a}\;. \end{array}$$ (51)

By forming an analogous Lagrangian we can calculate **x**_*b*_ and consequently $\hat{b}\left(t\right)$ and so forth.

### Variance

Estimating whether the system is healthy or malfunctioning on the base of its response *h*(*t*) by the following scalar products

$$\begin{array}{c} c_{a}=\langle \hat{a}\left(t\right),h\left(t\right)\rangle \end{array}$$ (52)

$$\begin{array}{cc} c_{b} & =\langle \hat{b}\left(t\right),h\left(t\right)\rangle \\ \vdots & \end{array}$$ (53)

is encumbered with an error. The sources of this error are measurement noise, stochasticity (non-repeatability) of the monitored process and differences in the training responses *a*_*i*_(*t*), *b*_*i*_(*t*), etc. Thus *c*_*a*_, *c*_*b*_ and so on can be treated as stochastic variables, for which we will calculate the variance. On the base of conditions (23–25), (26–28) we can calculate the unbiased estimator of the said variance

$$\begin{array}{c} \sigma_{a}^{2}=\frac{1}{\Theta -1}{\left(\langle \hat{a}\left(t\right),f\left(t\right)\rangle -v_{a}\right)}^{T}\;\left(\langle \hat{a}\left(t\right),f\left(t\right)\rangle -v_{a}\right) \end{array}$$ (54)

$$\begin{array}{rr} \sigma_{b}^{2} & =\frac{1}{\Theta -1}{\left(\langle \hat{b}\left(t\right),f\left(t\right)\rangle -v_{b}\right)}^{T}\;\left(\langle \hat{b}\left(t\right),f\left(t\right)\rangle -v_{b}\right)\\ \vdots & \end{array}$$ (55)

where, for the sake of simplicity, the result of $\langle \hat{a}\left(t\right),f\left(t\right)\rangle$ is understood as the following vector

$$\begin{array}{c} \langle \hat{a}\left(t\right),f\left(t\right)\rangle =\left[\begin{array}{c} \langle \hat{a}\left(t\right),f_{1}\left(t\right)\rangle \\ \langle \hat{a}\left(t\right),f_{2}\left(t\right)\rangle \\ \vdots \end{array}\right]\;. \end{array}$$ (56)

Parameters $\sigma_{a}^{2}$, $\sigma_{b}^{2}$, … will serve as measures for the goodness of fit to unseen data.

## Results

### Application

**Fig 5:**
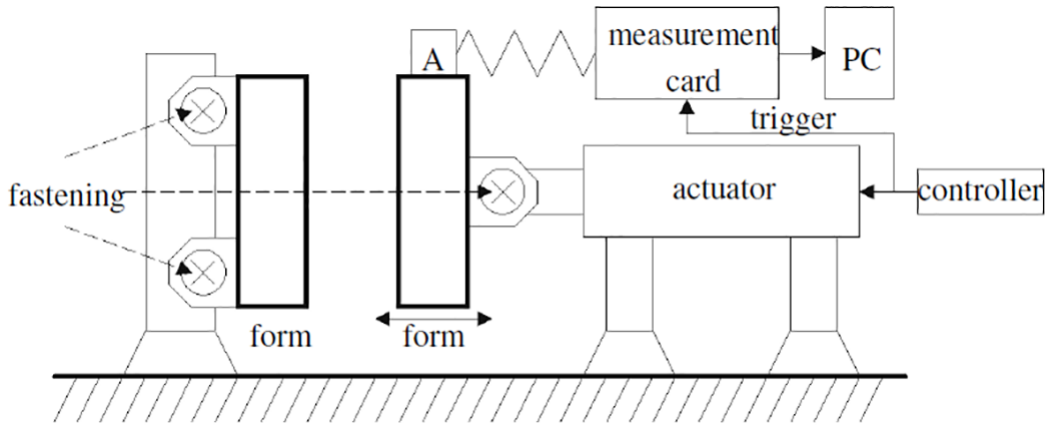
A simplified diagram of a moulding process being monitored. “A” stands for an accelerometer, “PC” for personal computer where the signals from the measurement card were stored and analysed.

The proposed method was applied to monitor a bottle moulding process. A simplified diagram of the device is presented in Fig 5.

The system is composed of a static and moveable form (die) where the latter is shifted by an actuator, till both dies meet. The forms are pressed against each other with strong force. Then, a preformed plastic bottle is blown and expanded into the inner shape of the forms.

**Fig 6:**
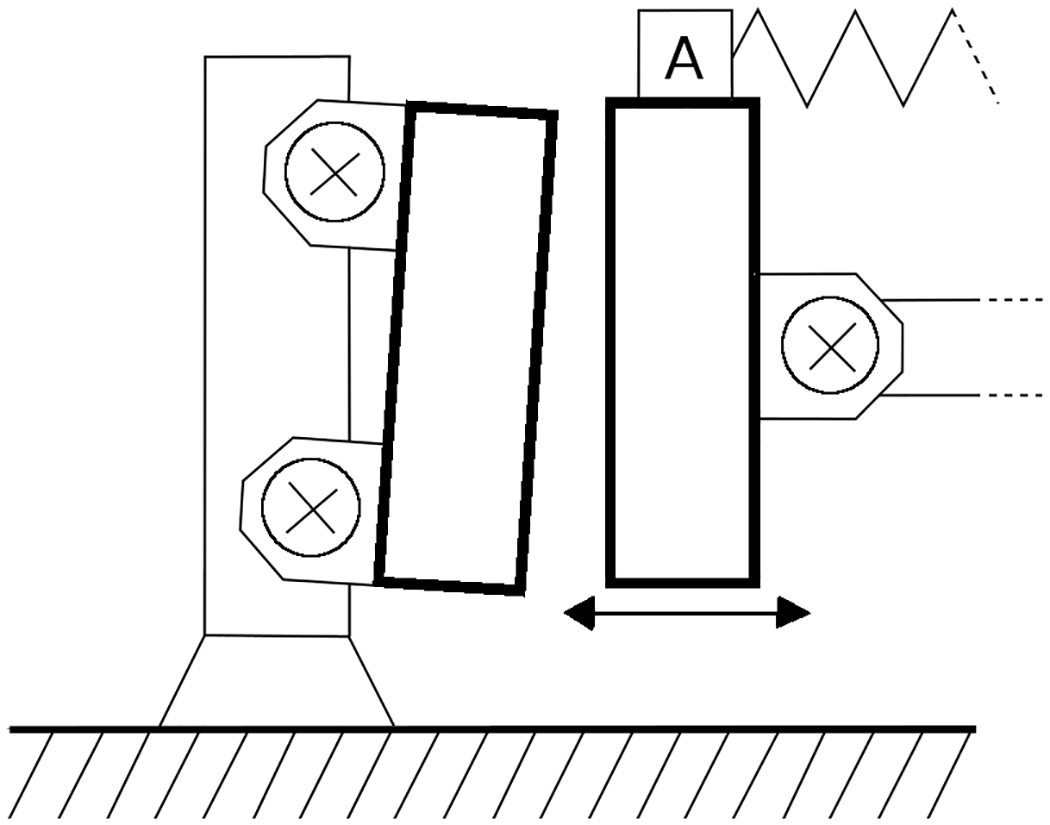
A sketch showing an exaggerated situation of misaligned forms.

The fastening screws provide proper alignment of the forms. Over time and due to high pressure, the fastening can become loose which results in an early degradation of the forms—an exaggerated situation is shown in Fig 6.

The second type of fault that occurred, albeit less important, was the loose fastening of the actuator to the body which caused additional vibration due to resonance.

### Measurements

**Fig 7:**
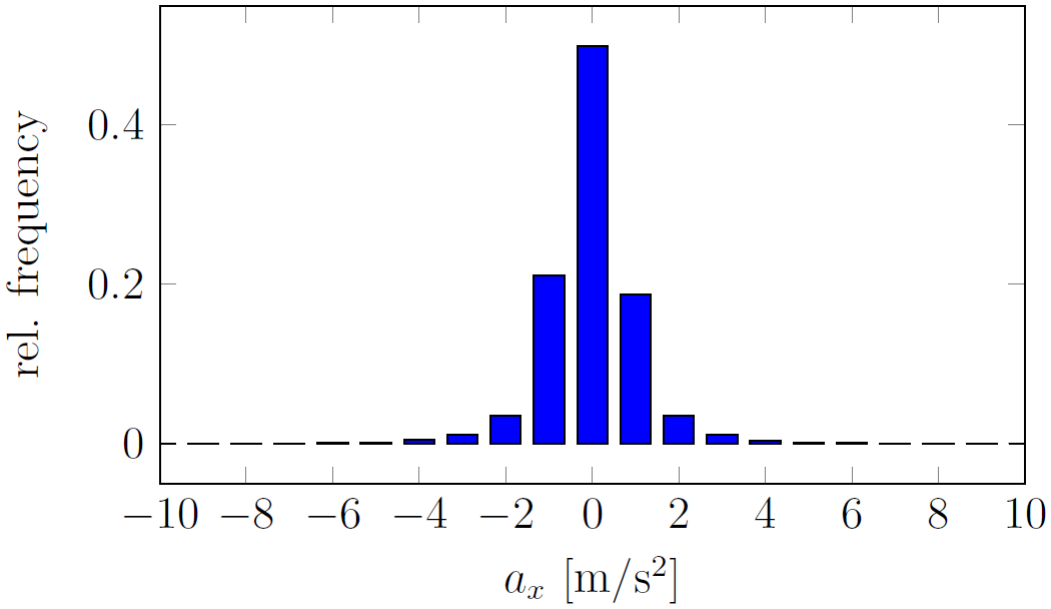
Histogram of the measurement noise (acceleration in a horizontal direction).

An analog piezoelectric accelerometer was placed on the moveable form and the acceleration signal along the axis of movement (horizontally) was registered. The parameters of the accelerometer were as follows:

- 3 dB bandwidth: 1 Hz to 10 kHz,
- acceleration range: ±100 *g*,
- sensitivity: 50 mV/*g*,

where *g* denotes the gravity of Earth, i.e. *g* ≈ 9.81 *m*/*s*^2^. The analog signal from the accelerometer was amplified, filtered and sampled at 65536 *Hz* by an acquisition card. Due to external noise, namely other machines in the factory producing vibrations, the accuracy of the measurements was degraded. The resultant noise was measured during the machine idle time and standard deviation equalled *σ*_*n*_ = 1.04 *m*/*s*^2^. The noise histogram is presented in Fig 7.

### Signature functions

**Fig 8:**
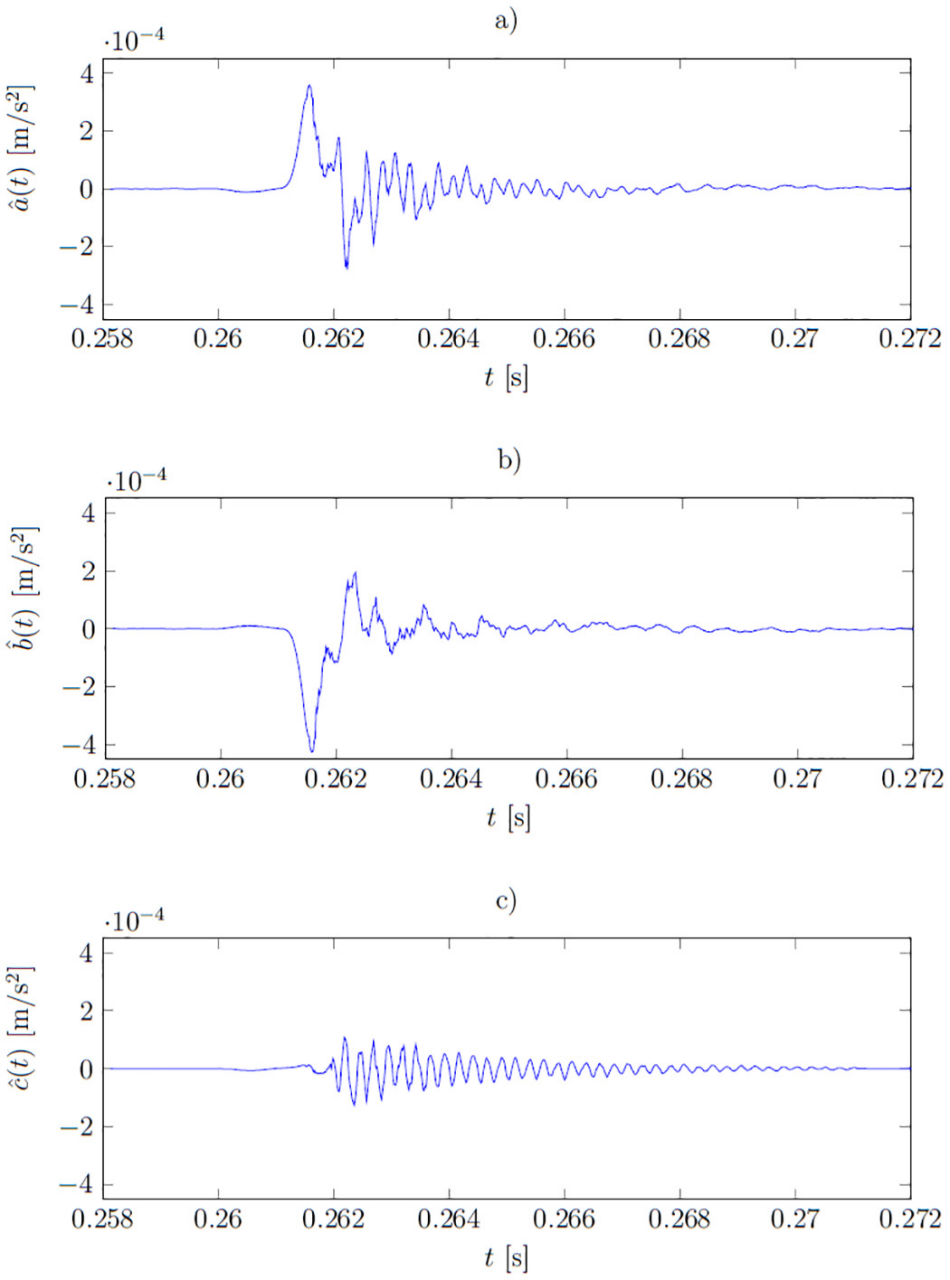
Plots of signature functions. a) under healthy conditions—$\hat{a}\left(t\right)$; b) under the first type of fault—$\hat{b}\left(t\right)$; c) under the second type of fault—$\hat{c}\left(t\right)$.

We measured 60 system’s responses—20 for each state (healthy, misaligned forms, loose fastening of the actuator). We divided this set into two parts: the training set

$$\begin{array}{cc} f(t) & ={\left[f_{1}\left(t\right),f_{2}\left(t\right),\ldots ,f_{18}\left(t\right)\right]}^{T}\\ & ={\left[a_{1}\left(t\right),\ldots ,a_{6}\left(t\right),b_{1}\left(t\right),\ldots ,b_{6}\left(t\right),c_{1}\left(t\right),\ldots ,c_{6}\left(t\right)\right]}^{T} \end{array}$$ (57)

containing 18 functions and the verification set

$$\begin{array}{cc} f_{v}\left(t\right) & ={\left[f_{v,1}\left(t\right),f_{v,2}\left(t\right),\ldots ,f_{v,42}\left(t\right)\right]}^{T}\\ & ={\left[a_{v,1}\left(t\right),\ldots ,a_{v,14}\left(t\right),b_{v,1}\left(t\right),\ldots ,b_{v,14}\left(t\right),c_{v,1}\left(t\right),\ldots ,c_{v,14}\left(t\right)\right]}^{T} \end{array}$$ (58)

containing 42 functions. Fig 3a, 3b and 3c show training signals measured when the system was calibrated, the forms misaligned and the actuator’s fastening screws became loose, respectively. The signature functions $\hat{a}\left(t\right)$, $\hat{b}\left(t\right)$ and $\hat{c}\left(t\right)$ of the healthy system and under two types of malfunctioning respectively, were calculated on the base of the training set **f**(*t*) and are presented in Fig 8. The verification set **f**_*v*_(*t*) is used to assess the results.

Table 2 shows the results of the standard deviations of *c*_*a*_, *c*_*b*_ and *c*_*c*_ (see Eqs (54 and 55)) calculated for the verification set of functions **f**_*v*_(*t*).

**Table 2:** Standard deviations of *c*_*a*_, *c*_*b*_ and *c*_*c*_ (for the verification set **f**_*v*_(*t*)) are calculated as *σ*_*a*_, *σ*_*b*_ and *σ*_*c*_—see Eqs (54 and 55).

*σ*_*a*_	*σ*_*b*_	*σ*_*c*_
0.146	0.134	0.056

For each function from the verification set **f**_*v*_(*t*), the following projections (see Eq (17)) into the signal space were calculated

$$\begin{array}{c} P\left(f_{v,i}\left(t\right)\right)=\left(\langle \hat{a}\left(t\right),f_{v,i}\left(t\right)\rangle ,\langle \hat{b}\left(t\right),f_{v,i}\left(t\right)\rangle ,\langle \hat{c}\left(t\right),f_{v,i}\left(t\right)\rangle \right)\;. \end{array}$$ (59)

**Fig 9:**
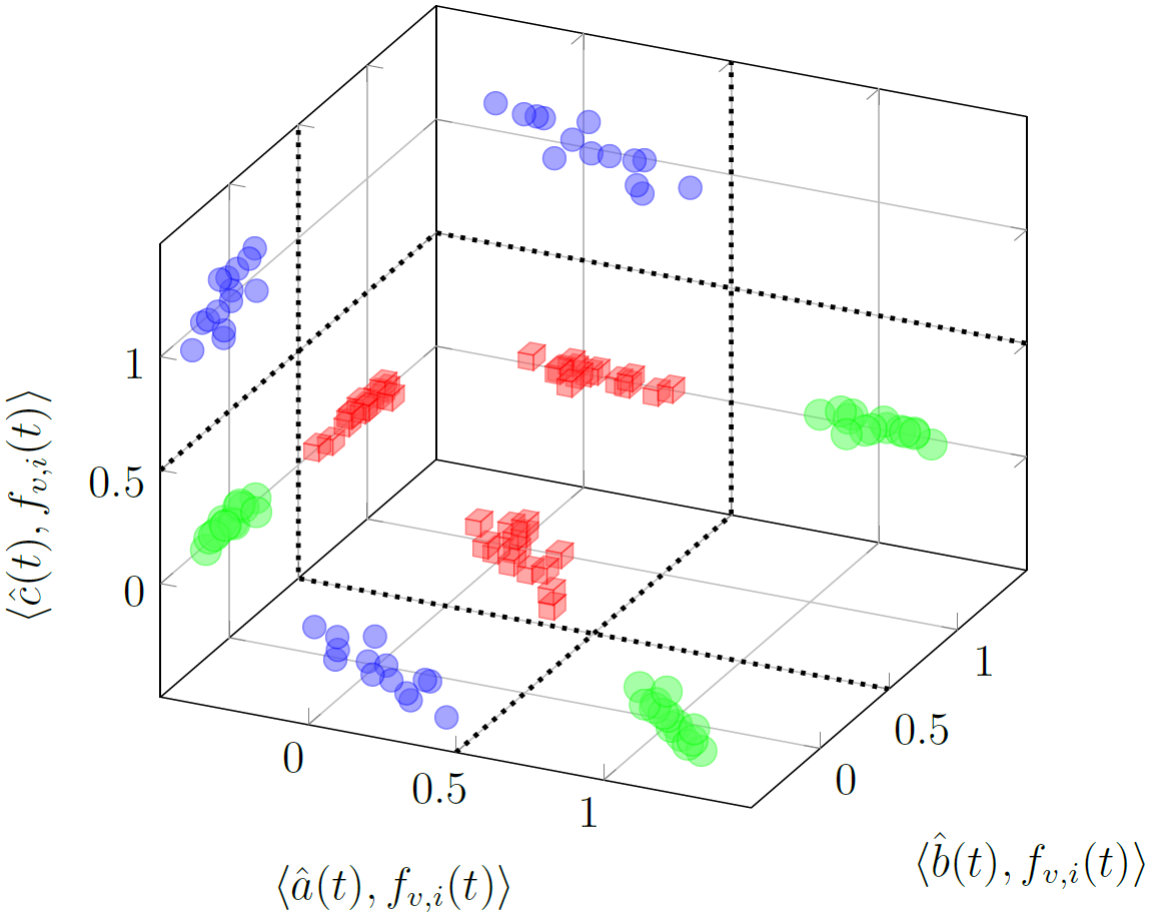
Projection of points *P*(*f*_*v*, *i*_(*t*)) for the functions from the verification set **f**_*v*_(*t*) on 3 planes. Big green spheres—projections of points *P*(*a*_*v*, *i*_(*t*)); red cubes—projections of *P*(*b*_*v*, *i*_(*t*)); small blue spheres—projections of *P*(*c*_*v*, *i*_(*t*)).

Points *P*(*f*_*v*, *i*_(*t*)) for the functions from the verification set **f**_*v*_(*t*) are visualized in Fig 9. The device’s state can simply be determined by analysing the location of the *P*(*h*(*t*)) point whereby *h*(*t*) is the current response. Perpendicular planes can be drawn to denote the boundaries for different conditions, which in the considered case, are easily distinguishable.

The algorithm using 32-bit fixed-point arithmetic was implemented on a simple ARM7 microcontroller (AT91SAM7x [23]) clocked at 50 *MHz*. The dot product of the signature functions $\hat{a}\left(t\right)$, $\hat{b}\left(t\right)$ and $\hat{c}\left(t\right)$ with the device response *h*(*t*) required ca 0.83 *ms* of the microcontroller’s time. There were only around 2750 fixed point multiplications and additions.

#### Overfitting

The problem of overfitting was mentioned earlier during the justification of the applied assumptions. As indicated before, the overfitted signature functions are calculated (on the base of the training set **f**(*t*)) from the following equations

$$\begin{array}{c} D\;x_{oa}=y_{a} \end{array}$$ (60)

$$\begin{array}{c} D\;x_{ob}=y_{b} \end{array}$$ (61)

$$\begin{array}{c} D\;x_{oc}=y_{c}\;. \end{array}$$ (62)

Solving for the unknown coefficients **x**_*oa*_, **x**_*ob*_, **x**_*oc*_ we obtain

$$\begin{array}{c} x_{oa}=D^{-1}\;y_{a} \end{array}$$ (63)

$$\begin{array}{c} x_{ob}=D^{-1}\;y_{b} \end{array}$$ (64)

$$\begin{array}{c} x_{oc}=D^{-1}\;y_{c} \end{array}$$ (65)

and finally the overfitted signatures equal

$$\begin{array}{c} \hat{\alpha }\left(t\right)=f{\left(t\right)}^{T}x_{oa} \end{array}$$ (66)

$$\begin{array}{c} \hat{\beta }\left(t\right)=f{\left(t\right)}^{T}x_{ob} \end{array}$$ (67)

$$\begin{array}{c} \hat{\gamma }\left(t\right)=f{\left(t\right)}^{T}x_{oc} \end{array}$$ (68)

where $\hat{\alpha }\left(t\right)$ is the signature under the system’s healthy condition; $\hat{\beta }\left(t\right)$ is the signature of the first type fault; and $\hat{\gamma }\left(t\right)$—of the second type fault. The plots of the overfitted signatures were presented in Fig 4. The overfitted functions are less smooth. The cause-observation relationship is obfuscated. For instance, at *t* ≈ 0.2605 *s* the signature $\hat{\beta }\left(t\right)$ assumes significant non-zero values, whereas the healthy *a*_*i*_(*t*) and faulty *b*_*i*_(*t*) responses differ significantly only after *t* > 0.2612 *s*—compare Figs 8 and 4.

Table 3 shows the results of the standard deviations of *c*_*a*_, *c*_*b*_ and *c*_*c*_ (see Eqs (54 and 55)) calculated for the verification set of functions. The results are worse than in the previous case (whereby $\hat{a}\left(t\right)$, $\hat{b}\left(t\right)$, $\hat{b}\left(t\right)$ signatures were used)—cf. Table 2.

**Table 3:** Standard deviations of *c*_*a*_, *c*_*b*_ and *c*_*c*_ (for the functions from the verification set **f**_*v*_(*t*) and overfitted signatures) are calculated as *σ*_*oa*_, *σ*_*ob*_ and *σ*_*oc*_—see Eqs (54 and 55).

*σ*_*oa*_	*σ*_*ob*_	*σ*_*oc*_
0.185	0.227	0.023

By analogy, we define the projections (see Eq (17)) for the overfitted signatures

$$\begin{array}{c} P_{o}\left(f_{v,i}\left(t\right)\right)=\left(\langle \hat{\alpha }\left(t\right),f_{v,i}\left(t\right)\rangle ,\langle \hat{\beta }\left(t\right),f_{v,i}\left(t\right)\rangle ,\langle \hat{\gamma }\left(t\right),f_{v,i}\left(t\right)\rangle \right)\;. \end{array}$$ (69)

**Fig 10:**
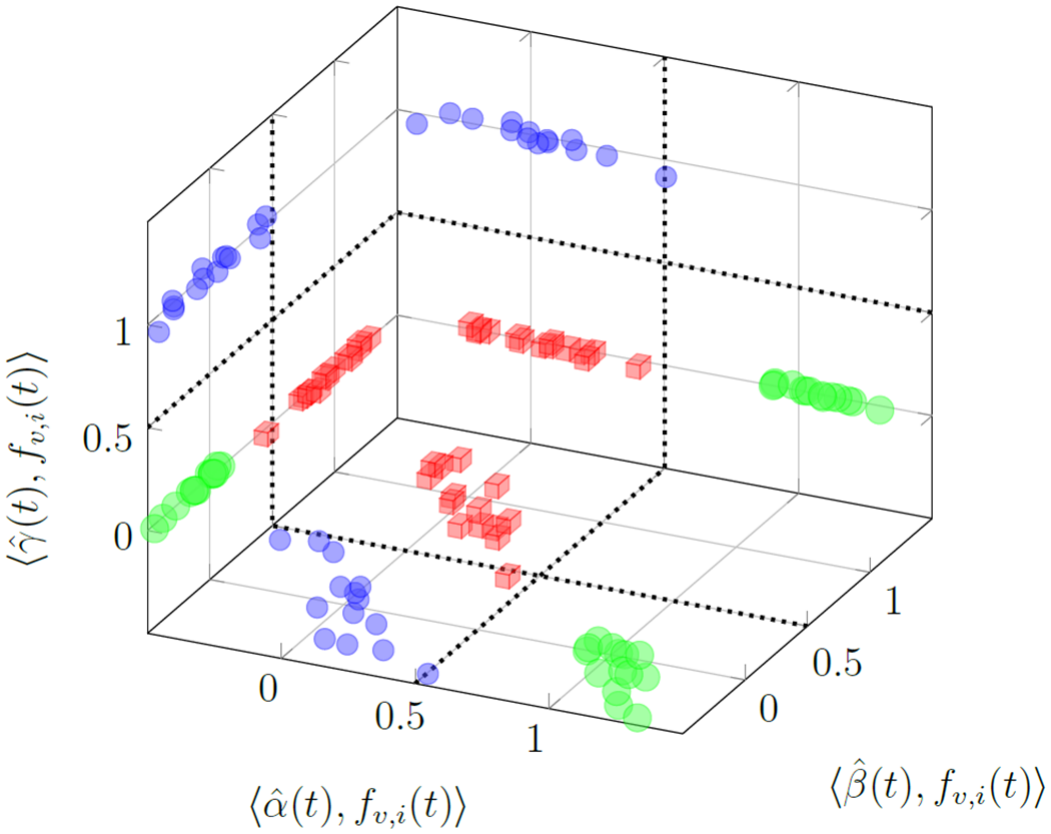
Projection of points *P*_*o*_(*f*_*v*, *i*_(*t*)) for functions from the verification set **f**_*v*_(*t*) on 3 planes. Big green spheres—projections of points *P*_*o*_(*a*_*v*, *i*_(*t*)); red cubes—projections of *P*_*o*_(*b*_*v*, *i*_(*t*)); small blue spheres—projections of *P*_*o*_(*c*_*v*, *i*_(*t*)).

Points *P*_*o*_(*f*_*v*, *i*_(*t*)) for the functions from the verification set **f**_*v*_(*t*) are shown in Fig 10. The *P*_*o*_(*a*_*v*, *i*_(*t*)) points are well fitted for the healthy state, however in the malfunctioning states the fit is poor and characterized by large variances—compare Tables 2 and 3. In this case, the decision boundaries cannot be represented by simple perpendicular planes. The points are more dispersed. Overfitting resulted in poor prediction for the verification responses.

## Discussion

A diagnosing method for an industrial moulding process of was presented, whereby the device was under three conditions: healthy, first and second type of fault. The solution proved to be effective in the considered application, where the device could be modelled as a stationary and linear system.

The solution can be adapted for different applications and expanded to diagnose multiple faults by creating corresponding signature functions, providing the stationary and linearity requirements are met.

The strong point of this approach is its simplicity for determining the system condition. The system response is projected into a signal space to determine its state. Thus, there is no need for a classifier such a neural network, hidden Markov models (HMM), support vector machine (SVM) for which the training phase can be a complex process.

Moreover, the underlying dot product operation is comparatively undemanding, as opposed to wavelet or Fourier transforms employed by other approaches. Therefore, a simple and low-cost processing unit can be applied to monitor an industrial process.

The presented solution can perform poorly when the responses are periodic with a greatly varying period or phase and also when the analysed system cannot be treated as stationary. In this case, the presented method can use results from the short time Fourier (STFT), wavelet transform, etc. to create multidimensional signature functions and benefit from a simplified classification. For example, employing STFT, 3 dimensional signature functions (amplitude, frequency and time) can be calculated. Such signatures can be robust against the above mentioned obstacles.

Another limitation occurs when one set of training functions is a scaled version of another function set. If this is the case, then an inconsistent system of equations is obtained, e.g. the following equations cannot be simultaneously satisfied:

$$\begin{array}{c} \langle \hat{a}\left(t\right),d\left(t\right)\rangle =1 \end{array}$$ (70)

$$\begin{array}{c} \langle \hat{a}\left(t\right),e\left(t\right)\rangle =\langle \hat{a}\left(t\right),k\cdot d\left(t\right)\rangle =k\cdot \langle \hat{a}\left(t\right),d\left(t\right)\rangle =0 \end{array}$$ (71)

where *k* is some constant different from 0. To detect these situations, the linear dependence of the training functions needs to be examined. The dimension of the signal space (i.e. number of signature functions) must be less than the total number of the system’s states. The problem needs to be slightly reformulated, however can be handled by the presented method.

A non-linear relationship between the faults and the output responses can compromise the solution, as the coexistence of faults does not correspond to the superposition of the responses under pertaining faulty conditions. The challenge of multiple fault diagnosis is difficult in general [24], however arises considerably less often than detecting a single failure.

## Supporting Information

S1 Dataset Measurement data. MATLAB files. Sampling rate: fs = 65536 Hz; a.mat—6 training functions for healthy state; b.mat—6 training functions for misaligned dies; c.mat—6 training functions for resonance; av.mat—14 training functions for healthy state; bv.mat—14 training functions for misaligned dies; cv.mat—14 training functions for resonance.
(ZIP)

## References

[1] Zhou C, Zhang W. A New Process Monitoring Method Based on Waveform Signal by Using Recurrence Plot. Entropy. 2015; 17: 6379–6396. 10.3390/e17096379

[2] Eckmann J, Kamphorst S.O, Ruelle D. Recurrence plots of dynamical systems. Europhysics Letters. 1987; 4 10.1209/0295-5075/4/9/004

[3] Ming G, Yangsheng X, Ruxu D. An Intelligent Online Monitoring and Diagnostic System for Manufacturing Automation. IEEE Transactions on Automation Science and Engineering. 2008; 5(1): 127–139. 10.1109/TASE.2006.886833

[4] Ge M, Du R, Xu Y. Hidden Markov Model based fault diagnosis for stamping processes. Mechanical Systems and Signal Processing. 2004; 18: 391–408. 10.1016/S0888-3270(03)00076-1

[5] Zhou S, Baocheng S, Shi J. An SPC Monitoring System for Cycle-Based Waveform Signals Using Haar Transform. IEEE Transactions on Automation Science and Engineering. 2008; 3(1): 60–72. 10.1109/TASE.2005.859655

[6] Ruiz-Gonzalez R, Gomez-Gil J, Gomez-Gil F, Martinez-Martinez V. An SVM-Based Classifier for Estimating the State of Various Rotating Components in Agro-Industrial Machinery with a Vibration Signal Acquired from a Single Point on the Machine Chassis. Sensors. 2014; 14: 20713–20735. 10.3390/s141120713 25372618 PMC4279508

[7] Liu Z, Liu Y, Shan H, Cai B, Huang Q. A Fault Diagnosis Methodology for Gear Pump Based on EEMD and Bayesian Network. PLoS ONE. 2015; 10(5): e0125703 10.1371/journal.pone.0125703 25938760 PMC4418566

[8] Lei Y, Lin J, He Z, Kong D. A Method Based on Multi-Sensor Data Fusion for Fault Detection of Planetary Gearboxes. Sensors. 2012; 12:2005–2017. 10.3390/s120202005 22438750 PMC3304152

[9] Tchakoua P, Wamkeue R, Ouhrouche M, Slaoui-Hasnaoui F, Tameghe T.A, Ekemb G. Wind Turbine Condition Monitoring: State-of-the-Art Review, New Trends, and Future Challenges. Energies. 2014; 7: 2595–2630. 10.3390/en7042595

[10] Cui J, Wang Y. A novel approach of analog fault classification using a Support Vector Machine Classifier. Metrology and Measurement Systems. 2010; XVII(4): 561–582

[11] Zhang C, He Y, Zuo L, Wang J, He W. A novel approach to diagnosis of analog circuit incipient faults based on KECA and OAO LSSVM. Metrology and Measurement Systems. 2015; XXII(2): 251–262.

[12] Kuczynski A, Ossowski M. Analog circuits diagnosis using discrete wavelet transform of supply current. Metrology and Measurement Systems. 2009; XVI(1): 77–84

[13] Majkowski A, Kolodziej M, Rak R. Joint time-frequency and wavelet analysis—an introduction. Metrology and Measurement Systems. 2014, XXI(4): 741–758.

[14] Tharrault Y, Mourot G, Ragot J, Maquin D. Fault detection and isolation with robust principal component analysis. International Journal of Applied Mathematics And Computer Science. 2008; 18(4): 429–442. 10.2478/v10006-008-0038-3

[15] Nowicki A, Grochowski M, Duzinkiewicz K. Data-driven models for fault detection using kernel PCA: a water distribution system case study. International Journal of Applied Mathematics And Computer Science. 2012; 22(4): 939–949. 10.2478/v10006-012-0070-1

[16] Paynabar K, Jin J, Pacella M. Monitoring and diagnosis of multichannel nonlinear profile variations using uncorrelated multilinear principal component analysis. IIE Transactions. 2013; 45: 1235–1247. 10.1080/0740817X.2013.770187

[17] Grzechca D. Soft fault clustering in analog electronic circuits with the use of self organizing neural network. Metrology and Measurement Systems. 2011; XVIII(4): 555–568.

[18] Bouthiba T. Fault location in EHV transmission lines using artificial neural networks. International Journal of Applied Mathematics And Computer Science. 2004, 14(1): 69–78

[19] Grzechca D, Rutkowski J. Fault diagnosis in analog electronic circuits—the SVM approach. Metrology and Measurement Systems. 2009; XVI(4): 583–598

[20] Shawe-Taylor J, Cristianini N. Kernel Methods for Pattern Analysis. Cambridge: Cambrigde University Press; 2004

[21] Clarke R, Ressom H, Wang A, Xuan J, Liu M, Gehan E, Wang Y. The properties of high-dimensional data spaces: implications for exploring gene and protein expression data. Nature Reviews Cancer. 2008, 1(8): 37–49. 10.1038/nrc2294 PMC2238676 18097463

[22] Brandt S. Statistical And Computational Methods in Data Analysis. 3rd edition New York: Springer Verlag; 1999

[23] Atmel’s website with the documentation of the AT91SAM7x microcontroller. Available: http://www.atmel.com/devices/SAM7X256.aspx

[24] Tadeusiewicz M, Halgas S. Multiple soft fault diagnosis of nonlinear DC circuits considering component tolerances. Metrology and Measurement Systems. 2011; XVIII(3): 349–360

